# Motile Male Gametes of the Araphid Diatom *Tabularia fasciculata* Search Randomly for Mates

**DOI:** 10.1371/journal.pone.0101767

**Published:** 2014-07-03

**Authors:** Robyn Edgar, David Drolet, James M. Ehrman, Irena Kaczmarska

**Affiliations:** 1 Département de biologie, Université Laval, Québec, Québec, Canada; 2 Department of Health Management, Atlantic Veterinary College, University of Prince Edward Island, Charlottetown, Prince Edward Island, Canada; 3 Digital Microscopy Facility, Mount Allison University, Sackville, New Brunswick, Canada; 4 Biology Department, Mount Allison University, Sackville, New Brunswick, Canada; Universidad Nacional Autónoma de México, Mexico

## Abstract

Sexuality in the marine araphid diatom *Tabularia* involves an unusual type of gamete, not only among diatoms but possibly in all of nature. The non-flagellated male gamete is free and vigorously motile, propelled by pseudopodia. However, the cues (if any) in their search for compatible female gametes and the general search patterns to locate them are unknown. We tracked and compared male gamete movements in the presence and absence of receptive female gametes. Path linearity of male movement was not affected by presence of female gametes. Male gametes did not move towards female gametes regardless of their proximity to each other, suggesting that the detection range for a compatible mate is very small compared to known algal examples (mostly spermatozoids) and that mate recognition requires (near) contact with a female gamete. We therefore investigated how male gametes move to bring insight into their search strategy and found that it was consistent with the predictions of a random-walk model with changes in direction coming from an even distribution. We further investigated the type of random walk by determining the best-fit distribution on the tail of the move length distribution and found it to be consistent with a truncated power law distribution with an exponent of 2.34. Although consistent with a Lévy walk search pattern, the range of move lengths in the tail was too narrow for Lévy properties to emerge and so would be best described as Brownian motion. This is somewhat surprising because female gametes were often outnumbered by male gametes, thus contrary to the assumption that a Brownian search mode may be most optimal with an abundant target resource. This is also the first mathematically analysed search pattern of a non-flagellated protistan gamete, supporting the notion that principles of Brownian motion have wide application in biology.

## Introduction

In complex, heterogonous environments, mobile organisms are confronted by the daunting task of finding resources (food, host, sexual partner, etc.) necessary for sustainability of the population. To do so they use a wide variety of cues to locate target resources from a distance. However, these cues can typically only be detected within a specific range. In the absence of cues, various types of random movement may be used to increase the probability of an encounter [1], [2], [3]. The optimal search strategy seems to be dependent on the abundance and distribution of the target resource [3], [4], [5]. When it is sparse, highly directed movement (e.g. correlated random walks or ballistic movement) may represent a good strategy. If the resource aggregates in patches, clusters of small test-moves interspersed by very long movements (Lévy walk), may be optimal for improvement of individual fitness [6]. On the other hand, when the target is abundant and randomly distributed, a series of randomly oriented moves (with little variation in move length, i.e., Brownian motion) may be optimal [5]. Individuals may switch from one search strategy to another in response to a change in the environment [7], [8], [9].

Although sensing environmental cues (e.g., nutrients, appropriate mate) is inherent to species ecology, it is a nascent field in diatom experimental ecology. To our knowledge only a few such studies have been published thus far, and exclusively for raphid pennates. One such work involved a motile, raphid pennate diatom [10] which demonstrated positive chemotaxis to specific sugars, similar to that observed in a number of green and other brown algae [11], [12]. For fertilization to occur, gametes have to meet and also recognize each other as compatible partners. Among diatoms, flagellated sperm in centrics are credited with the capacity to move towards an immotile egg [13]. In raphid pennate species, sexualized motile gametangia find each other and pair, usually following sex-induction cues [14] and within a specific cell-size range [15]. Recently, the behavioural, directional response to identified chemical cues exuded by a sexual partner was documented for a species of *Seminavis*, a motile raphid pennate [16], joining a relatively small volume of work of well-documented cases from other brown macroalgae [12] and various microalgae [17]. In contrast to other protists, including a few microalgae, the movement pattern of cells responding to chemical stimuli (including compatible mates) is yet to be analyzed mathematically for any diatom.

*Tabularia fasciculata* (C.A. Agardh) D. M. Williams and Round is one of only a few araphid pennate species known to produce vigorously motile, free, non-flagellated male gametes [18], [19], [20]. Male gamete motility in two *Tabularia* species coincides with extrusion and retraction of pseudopodia, thereby making gametes capable of spinning, shuffling and making jump-like displacements over a distance much greater than the diameter of the gamete [18], [19]. Therefore, they are a good candidate to investigate the male gamete search method for locating a receptive sex-partner. In largely immotile vegetative cells of araphids, parents-to-be are believed to be passively carried to the vicinity of each other by water turbulence. The absence of flagellated sperm and inherent immotility of parental gametangia of most of the araphid pennate species would seem to place the prospects for syngamy for araphid diatoms at a disadvantage relative to centrics and raphid pennates.

Therefore we compared the movement of male gametes in presence and absence of stationary female gametes to determine whether males can detect and consequently orient themselves towards potential mates. After determining they could not, we evaluated what type of search strategy may be used by males to enhance the probability of their encounter with a compatible female gamete. The significance of our results is discussed in the context of diatom reproductive and evolutionary biology.

## Methods

### Study organism and its sexual reproduction

*Tabularia fasciculata* is a benthic diatom typically growing on filaments or blades of small intertidal macroalgae. It is a colonial species forming pin-cushion clusters of needle-shaped cells [21]. Its sexual reproduction is primarily heterothallic [22]. The entire process and cells involved are described in [18]. Male and female gametogeneses and syngamy are readily inducible [21] and were observed numerous times in this and other studies. Two gametes are produced by each male and female gametangium [18], each gamete with sex-specific morphology and behavior. Male gametes are released into the environment where they can move freely and often vigorously by means hitherto unknown among diatom gametes and possibly in any gamete in general [18], [19]. Female gametes stay within maternal thecae that are attached to the substratum (mating well bottom) throughout auxosporulation. Fertilization results in a zygote, which elongates (becoming an auxospore) more or less parallel to the maternal theca, eventually producing a progeny initial cell with a large frustule. We routinely observed all these stages in the course of our experiments, confirming that gamete behavior and resulting zygotes observed in this study conform to the normal sexual process for this species. A small but statistically significant difference in mean male gamete diameter (female absent - 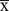 = 11.06 µm, SD = 0.90 µm, n = 37; female present -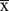 = 10.58 µm, SD = 0.98 µm, n = 33; t-statistic = 2.17, P = 0.03) was most likely due to vegetative cell diminution (and consequently gamete size [23]) over the year-long course of experiments. Measurements involving female gametes in proximity to male gametes were more frequent in the latter half of the experiments, thus involving more slightly smaller gametes.

### Laboratory manipulation, cell tracking and movement recording

The three clones of *Tabularia fasciculata* used in this study were isolated using micropipetting methods as described in [21]. The clone 507A1 was isolated from seawater collected at Cape Tormentine, NB, Canada (45°05′27″N, 66°28′16″W; 5 May, 2010), while 712A (formerly 612A) was isolated from a clump of seaweeds at Gleason Point Bar, Maine, USA (44°58′23″N, 67°03′13″W on 4 July, 2010). Clone 325A was the F1 progeny of crossing between TF0206A (female) × TF0206M (male) in March 2006 [24]. All three clones are genetically and morphologically characterized in [25].

Stock cultures were grown at 11–14°C at approx. 13 µmol photons m^−2^s^−1^ provided by Osram Sylvania Gro-Lux fluorescent lamps (Danvers, MA, USA) in 125 mL flasks in f/2 growth media at 6∶18 hours light:dark (L:D) cycle; irradiance measured with a LI-COR LI 250 photometer (Lincoln, NE, USA). Stock cultures were transferred weekly to maintain exponential growth phase. Immediately prior to mating, the flasks with newly transferred cells of parental clones were first kept for one day in the same growth conditions as the stock described above. The flasks were then moved to a growth cabinet at 18°C under 12∶12 hours L:D cycle with 34 µmol photons m^−2^s^−1^ provided by Osram Sylvania Cool White fluorescent lamps (Danvers, MA, USA) for approximately 5 days. Parents were then mixed together in pair-wise fashion in 9 replicate wells and two control wells each with only one parent clone in 12-well trays. These trays were placed back in the 6∶18 L:D cycle, 13–14°C growth chamber, as a change in ambient temperature induced sexuality. Terminology associated with sex cells and processes follow [22].

Reconnaissance mating experiments showed that the optimal time for gamete movement observation was after 48 hours incubation of mating parents in the growth chamber. Crossing pair 507A1×712A was the most successful of all of the pairs tested; 9.36% of cells showed mating activity and these were most often recorded, although other clone gametes were also examined and used to confirm that observed behavior was not pair specific. Parental cell size ranges varied depending on the clone and were: 55–75 µm for 507A1, 35–40 µm for 712A and 13.4–24.0 µm for 325A.

Live gametes were observed directly with a Zeiss Axiovert 200 inverted light microscope (Carl Zeiss Canada Ltd., Toronto, Canada). Male gamete movement paths were recorded as a time-lapse video captured using a QImaging MicroPublisher 3.3 RTV camera and its QCapture Pro software (v.5.0.1.26; QImaging, Surrey, Canada). Each video capture consisted of 50 frames, each 15 seconds apart, viewed through a 20x objective lens for the gametes that remained within the focal plane of this objective (5.8 µm depth of field) throughout the recording. Given the typical diameter of the observed gametes, this criterion essentially restricted data collection to gametes moving in two dimensions. Occasional recordings where the male gamete moved out of the focal plane (up or down in the well medium) were disregarded. An image of a larger field of view (using a 10x objective) was taken immediately prior to video recording to record the cell layout to be used as a reference for the start positions of all sex cells involved. Only one video was captured per well to minimize photic exposure of the gametes. Positions of cells, determined as X, Y pixel coordinates in digital video frames, were converted to positions expressed in micrometers (with 0,0 in the upper left of the image) by calibrating images at both magnifications using a stage micrometer with 10 µm spacing. The most vigorously motile male gamete was selected in each recording to represent performance with the greatest potential for syngamy because the age of individual male gametes (and possibly age-dependant vigour) could not be measured.

### Analysis of recordings

**Figure 1:**
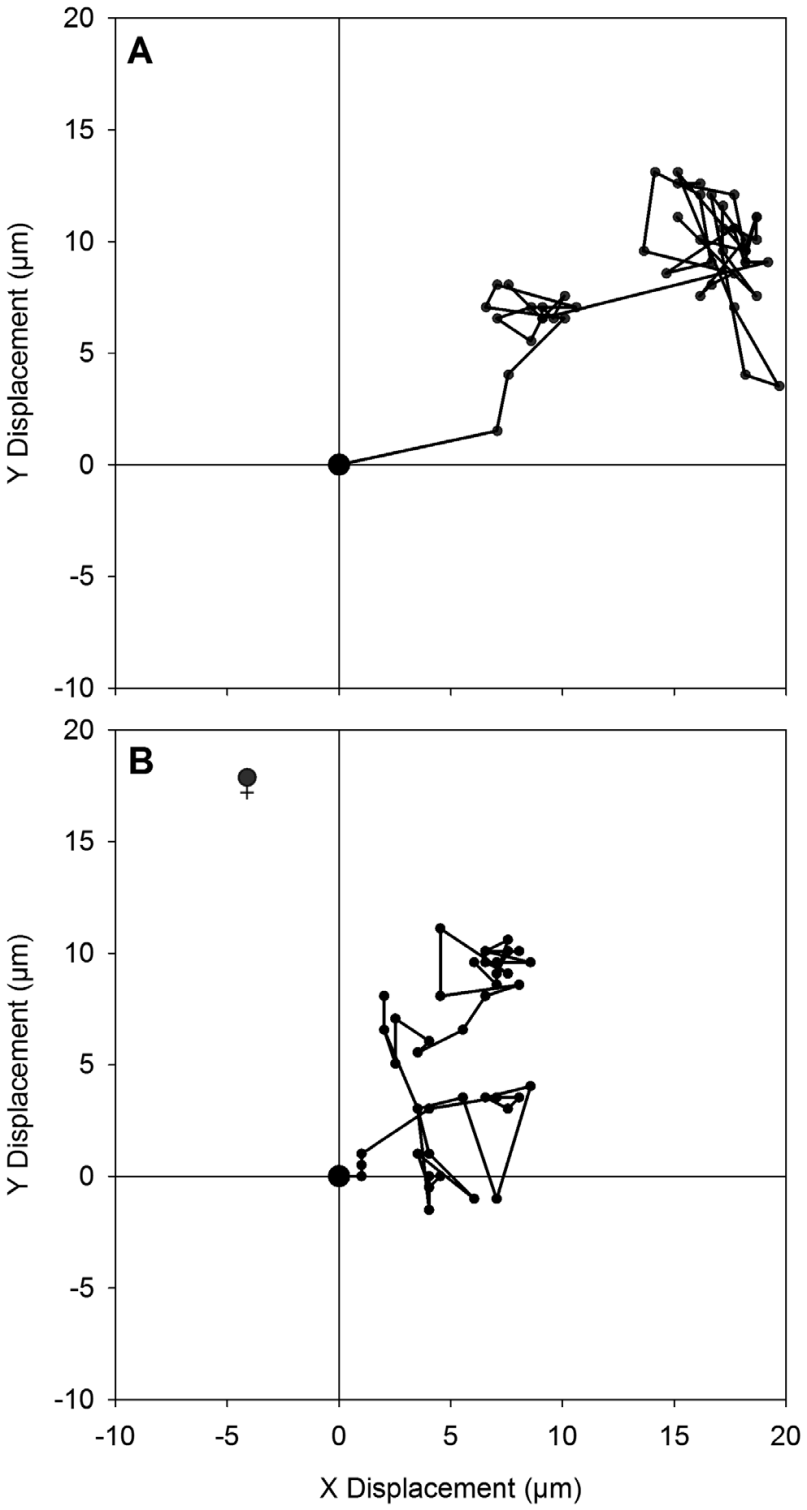
Representative trajectories of observed male gametes in the absence and presence of a receptive female gamete. A) Trajectory of male gamete in the absence of receptive female gamete. B) Trajectory of male gamete in the presence of receptive female gamete. Position of receptive female gamete indicated by ♀. Plots represent position of male gamete at each of the 15 second intervals of time-lapse imaging (50 frames over 12.5 minutes). Velocity for the particular male gamete in A) ranged between 0.03–0.69 µm s^−1^ and in B) between 0–0.35 µm s^−1^, although velocity variation between the two treatment groups collectively was not statistically significant. The sign of the x-values in A) and the y-values in B) have been changed so that trajectories are in a similar orientation and directly comparable.

**Figure 2:**
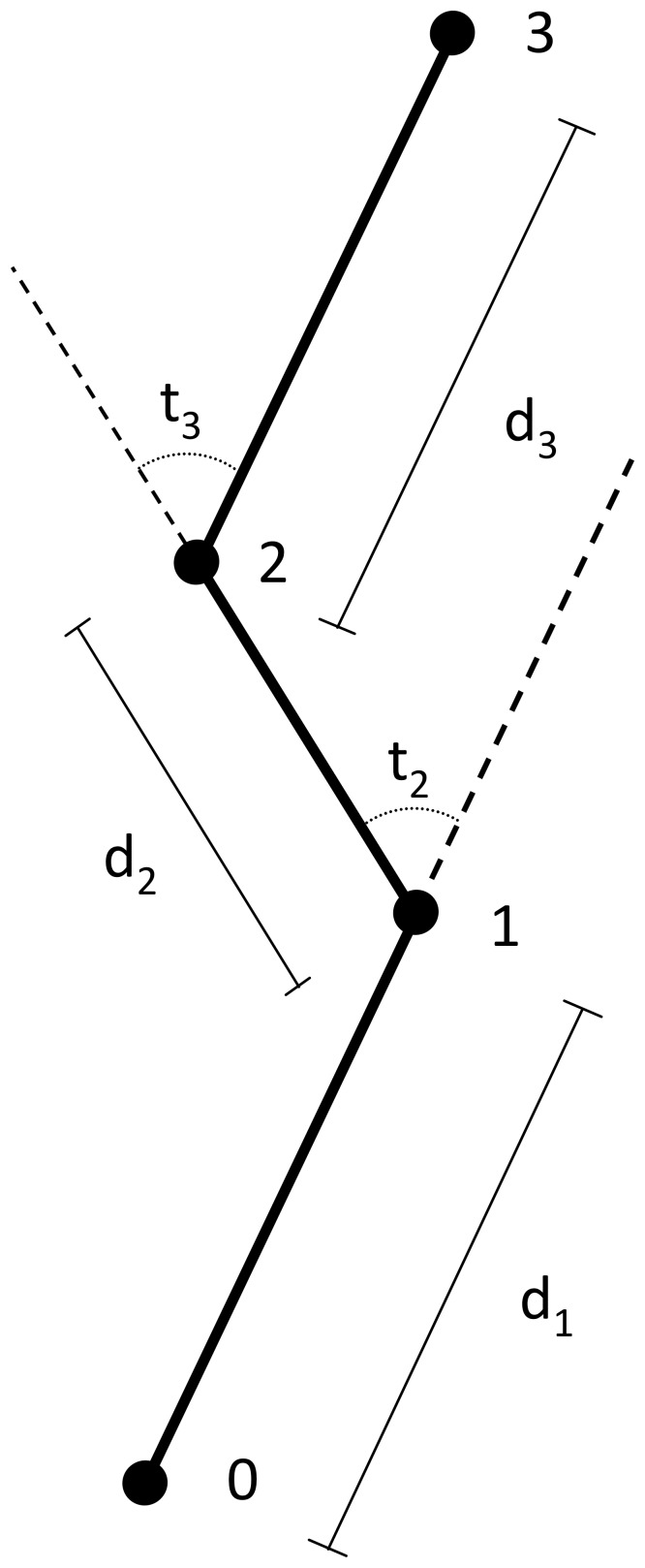
Schematic of a fictitious path of a male *Tabularia fasciculata* gamete and associated measurements. Dots represent position of the gamete at one-minute intervals, d_t_ shows the move lengths, and t_t_ shows turning angles.

A total of 70 videos were recorded under two conditions: 1) 37 male gamete movements in the absence of a non-fertilized female gamete/gametangium in the 10X objective field of view and 2) 33 male gamete movements in the presence of a non-fertilized female gamete/gametangium in the same size field of view. Representative trajectories for each experimental condition are shown in Fig. 1 (see also Videos S1, S2, and those in [19]). In the first case the minimum distance to a possible female cell was taken to be the distance from the male cell to the nearest edge of the field of view (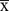 = 193.6 µm, SD = 59.9 µm, n = 37) and in the second case the actual distance between the two cells was measured (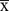 = 107.5 µm, SD = 114.4 µm, n = 33). ImageJ (v. 1.46f, National Institutes of Health, Bethesda, MD, USA) was used to calculate the coordinates of the center of the cell being tracked in each video frame. Steps and turning angles were calculated using these coordinates for every fourth frame (one minute intervals) in order to avoid oversampling [2]. Each step was calculated using the Pythagorean distance formula with the current frame and previous frame coordinates to obtain the distance traveled from the previous point (Figure 2). The turning angle was calculated within a range from [−180°, 180°]. The turning angle was measured as the difference between the direction the cell traveled during the previous time interval and the direction for the current time interval (Figure 2). If the turn was clockwise from the starting direction, the angle fell within the range [−180, 0]. If the turn was counter clockwise from the starting direction, the angle was within the range [0,180].

### Statistical analyses

We analysed male gamete velocity using an ANOVA with displacement per minute (µm) as the dependent variable. The independent variables included the fixed factor ‘Presence of female gamete’ (present or absent) and the random factor ‘Individual’ (the individual selected from each video sequence) nested in ‘Presence of female gamete’. Velocities were log10-transformed to correct for problems with the assumption of homogeneity of variances. We computed a linearity index defined as the ratio of net displacement (distance between starting and end point) and gross displacement (sum of distances traveled within each minute step). The index ranges from 0 to 1, with values close to 0 corresponding to very convoluted paths and values close to 1 corresponding to a directed movement. We also compared the linearity index (known also as the McCutcheon Index [26]), in relation to presence of female gamete with a t-test. These analyses were conducted using SPSS 19 (IBM Corp., New York, USA).

To determine whether male gamete movement was directed towards female gametes, we conducted an analysis of orientation. We first determined the direction that would lead the male gamete to the closest female gamete, i.e. the angle of a vector with the starting position of the male gamete and the position of the stationary female gamete as coordinates. We then determined the net direction of the male gamete movement, i.e. the angle of a vector with the starting and final position of the male gamete as coordinates. The deviation between direction of male gamete movement and the ‘target’ (i.e. female gamete) was calculated as the difference between the angles of the two vectors. We then conducted a Rayleigh test on the mean vector [27] to determine if the direction of male gamete movement, in relation to position of the female gamete, deviated from a random orientation. Finally, to determine if proximity of a female gamete influences the direction of male gametes, we conducted a linear regression analysis with absolute deviation (absolute value of the deviation described above) as the dependent variable and distance between the male and female gametes at the start of a video as the independent variable.

We compared the realized paths with the predictions of random-walk models in which the direction of a step is independent of the direction of the previous step. This was done to evaluate the possibility that gametes use correlated random walks, i.e., walks in which direction of the previous step influenced the direction of a next step. This would result in superdiffusivity and would be detected by a poor match between predictions of a random walk model and observed displacements. A model was fitted for each of the two treatments (female gamete present or absent). The expected net square displacements were calculated by generating random paths. A path consisted of 12 steps describing the position of gametes at one-minute intervals. The distance moved during each step was randomly selected from the empirical distributions obtained from the videos. The direction of the first move was randomly drawn from an even distribution of values ranging from −180 to 180°. The direction of subsequent moves was determined by randomly drawing a turning angle coming from the same distribution. We then computed the net square displacement (squared distance from the initial position) after each step. We generated 1000 random paths for each treatment and calculated the mean net square displacement at one-minute intervals. We also computed 95% confidence intervals around the mean expected net square displacements by retaining the 2.5^th^ and 9.75^th^ percentile for each step. We compared the predicted and realised net square displacements for the two treatments separately. These analyses were conducted using Matlab r2012b (MathWorks, Natick, MA, USA).

Inferences regarding the type of movement used by an individual can be made by examining its movement path and usually involves analysis of the shape of the tail-end of the distribution of move length (e.g., distance travelled between reorientation or distance travelled within fixed time intervals). Distributions with finite variance (e.g., exponential) are indicative of Brownian motion, such as movement shown by inanimate particles, bacteria, etc. Recently however, more research attention has been given to move-length distributions with power-law tails, particularly those with probability density functions defined by *f(x) = Cx^−µ^*, for *x>x_min_*, (where *x* is move length, *C* is a constant, and *x_min_* is the value of *x* where the distribution tail starts); such as movements observed among some foraging animals. After estimating the exponent *µ*, ballistic movement may be inferred if *µ* is close to one, Lévy walk if 1< *µ* <3, and Brownian motion if *µ* >3 [28]. To gain deeper insight into what specific search strategy is used by male gametes to locate female gametes, we evaluated what type of distribution best fit the tail of the empirical distribution of move lengths. The minimum move length at which the distribution tail started was determined following [29]. We tested four common distributions: 1) power law, 2) exponential, 3) bounded power law, and 4) bounded exponential. Parameters were estimated with maximum likelihood methods, following [30], [31]. Akaike Information Criteria were used to determine which distribution approximated the observed distribution best; we used the methods and code provided in [32]. Finally, we used a Kolmogorov-Smirnov goodness-of-fit test [27] to determine if the top-ranked distribution provided an appropriate fit to the observed data.

## Results

### Male gamete movement pattern

**Figure 3:**
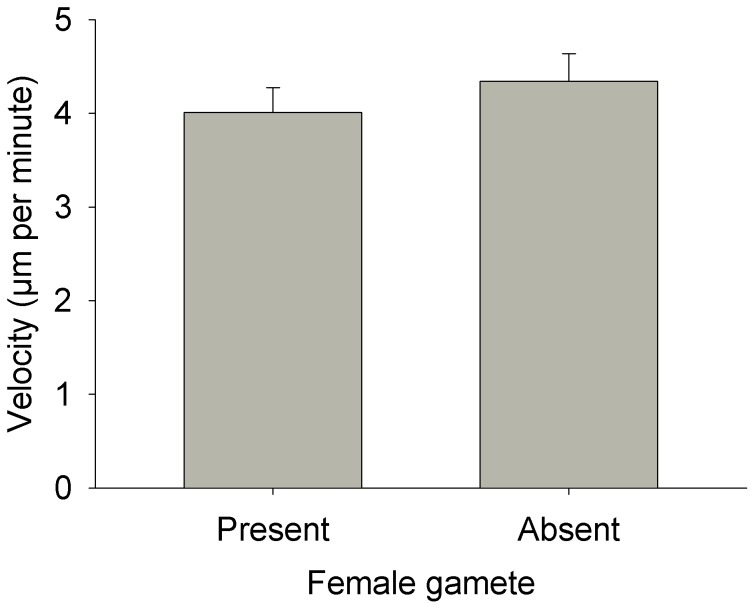
Mean (+SE) displacement velocity of *Tabularia fasciculata* male gametes. There was no statistical difference between the two treatments (presence/absence of female gamete). Analysis was conducted on log10-transformed data, but raw values are presented.

**Figure 4:**
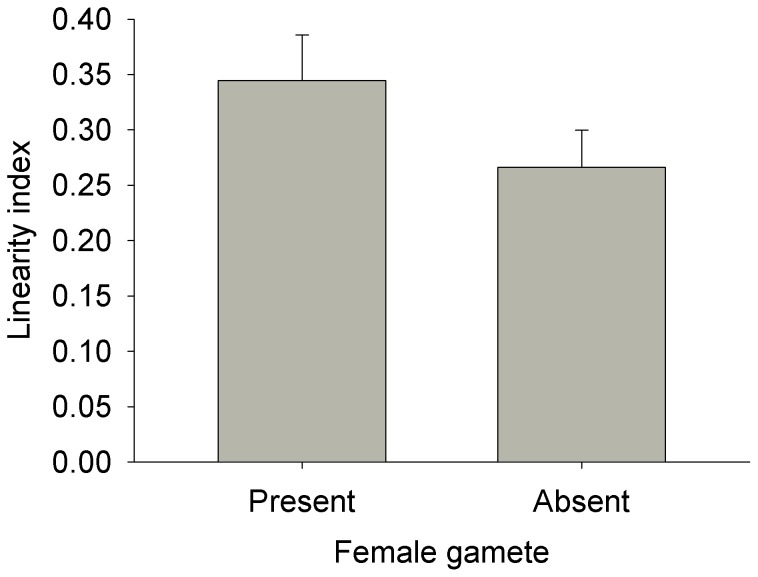
Mean (+SE) linearity index of movement in *Tabularia fasciculata* male gametes. There was no statistical difference between the two treatments (presence/absence of female gamete).

**Figure 5:**
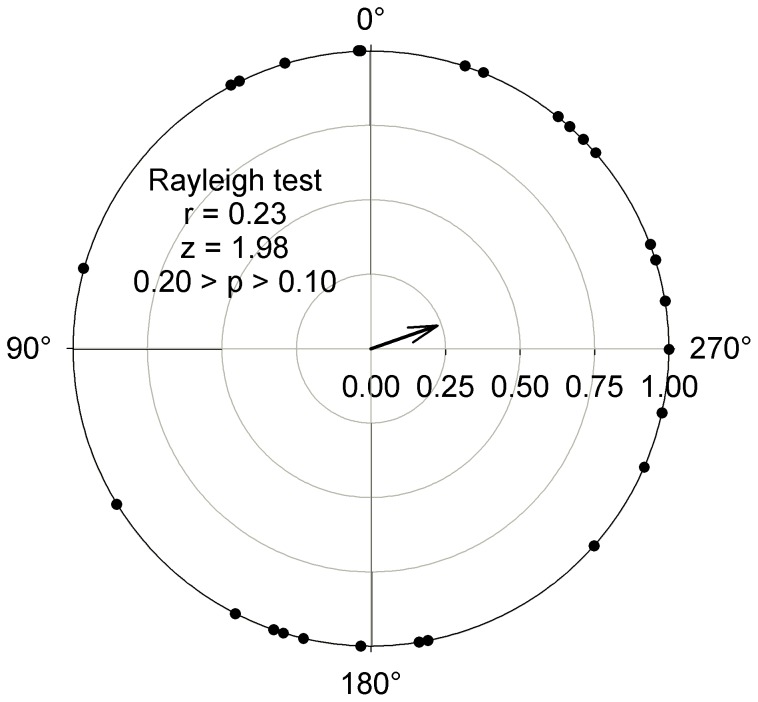
Deviation between *Tabularia fasciculata* male gamete movement direction and position of female gamete. A movement perfectly oriented towards the female would have a deviation of 0°. The arrow presents the length and direction of mean vector. Deviations were not different from a random distribution.

**Figure 6:**
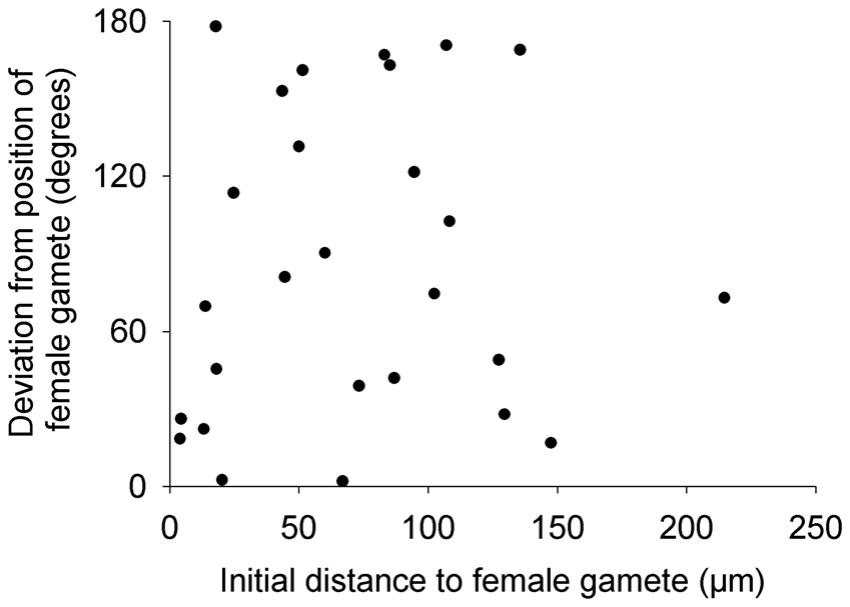
Absolute deviation between *Tabularia fasciculata* male gamete movement direction and position of female gamete. Deviation calculated in relation to distance between the female gamete and the starting position of the male gamete. The linear regression coefficient was not significant.

In general, male gamete movement was not influenced by the presence of a female gamete. Velocity varied among individuals (Nested ANOVA: MS = 0.44, F_68, 770_ = 5.88, p<0.0001) simply because some individuals were more active than others. This among-individual variation was not related to presence or absence of a female gamete (Nested ANOVA: MS = 8.1×10^−3^, F_1, 68_ = 0.02, p = 0.89; Figure 3). Similarly, the linearity indices did not vary in relation to presence or absence of female gametes (t-test: t_68_ = 1.51, p = 0.14; Figure 4). When in the presence of a female gamete, male gametes did not orient themselves towards the female. The deviation between the male gamete’s orientation and the position of the female gamete did not differ from random (Rayleigh test: z = 1.98, 0.2>p>0.1, n = 27; Figure 5) and the mean vector was oriented perpendicular to the position of the female gamete. Not all paths were used for this test because we excluded those where the female was present in the 10X field of view but not within the 20X field of view. Finally, the absolute deviation did not vary in relation to distance between the male and female gametes (linear regression: MS = 821.92, F_1,26_ = 0.23, p = 0.64, r^2^ = 0.009; Figure 6).

**Figure 7:**
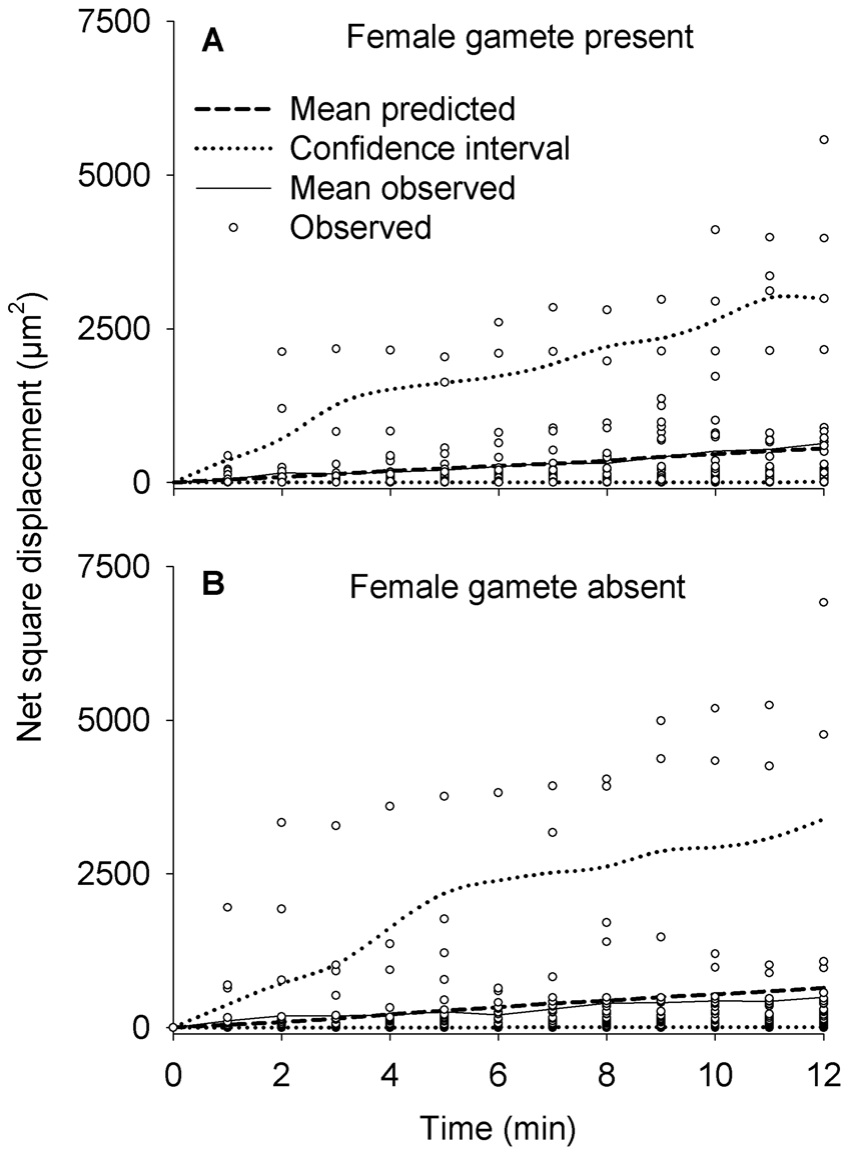
Realised versus predicted paths of *Tabularia fasciculata* male gametes. A) Female gamete present. B) Female gamete absent.

**Figure 8:**
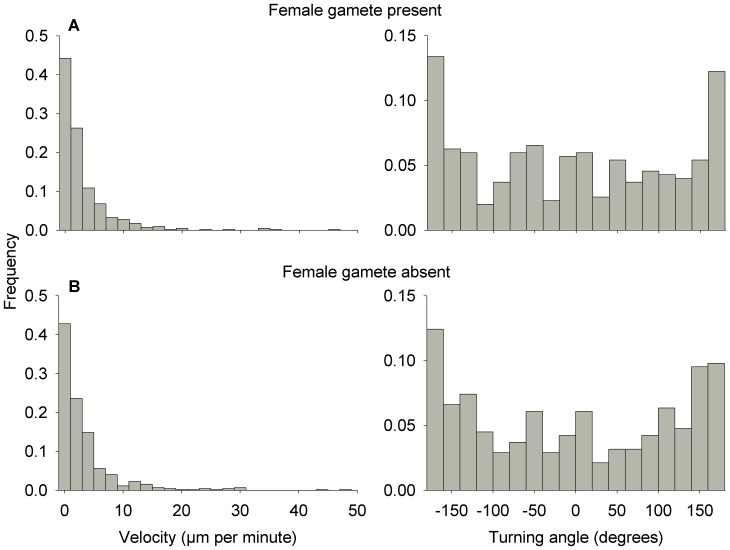
Frequency distribution of turning angles and velocity of *Tabularia fasciculata* male gametes. A) Female gamete present. B) Female gamete absent.

**Figure 9:**
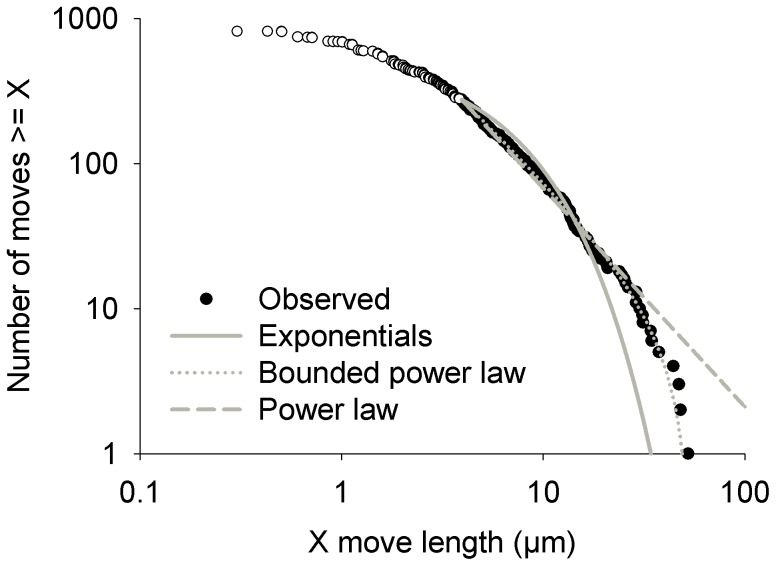
Rank frequency plot of move lengths of *Tabularia fasciculata* male gametes. Videos with presence and absence of a female gamete have been combined for this plot. The open circles represent moves not included in the tail of the distribution, whereas filled circles are part of the tail. The different lines represent the fit of the distributions tested. Note that the exponential and bounded exponential distributions overlap perfectly within the range of values observed.

The predicted and realised net square displacements of male gametes were very similar (Figure 7). Whether a female gamete was present or not, the mean realised net square displacement increased linearly over time and was almost identical to the mean prediction. All measured paths were within the confidence intervals of the random-walk models, with the exception of a few individuals that made a sudden long step within their otherwise compliant paths. The model predictions and realised paths were very similar whether a female gamete was present or not (Figure 7). Paths of male gametes consisted of periods of low levels of activity followed by sudden long distances travelled, resulting in distributions of velocities in which slow movements are very common and faster movements (up to ∼50 µm per minute) occur sporadically (Figure 8). The frequency distribution of turning angles showed that the most common changes in direction were close to 180°, in other words a complete reversal in direction (Figure 8). However, these frequency distributions did not appear to vary in relation to presence of a female gamete. The tail of the distribution of move lengths was determined to start at 3.93 µm and spanned over slightly more than one order of magnitude. It was better approximated by a truncated power law distribution (with an exponent of 2.34) than by any of the alternatives tested (Table 1). The truncated power law provided a very good approximation of the data (Figure 9) and there was no difference between the observed and expected cumulative density functions (Kolmogorov-Smirnov test, p = 0.90).

**Table 1:** Summary of distribution fitting to the tail of the empirical distribution of move lengths of male gametes (pooled across presence of female gamete treatments).

Distribution	Parameters	AIC	Likelihood
Truncated power law	µ = 2.34, x_max_ = 52.22	2096.92	0.997
Power law	µ = 2.51	2108.66	2.81E-3
Truncated exponential	λ = 0.06, x_max_ = 52.22	2145.79	2.42E-11
Exponential	λ = 0.06	2147.72	9.28E-12

Distributions (and estimated parameters) listed in decreasing likelihood of best fit. AIC = Akaike Information Criterion.

µ = estimated exponent of power law distributions with probability density function given by *f(x) = Cx^−µ^*, for x_max_ *>x>x_min_* for truncated power law and ∞>*x>x_min_* for power law distribution.

λ = parameter for exponential distribution with probability density function given by *f(x) = Ae-* ^λx^ for x_max_ *>x>x_min_* for truncated exponential and *f(x) = *λe_− λ(x-xmin)_ for ∞>*x>x_min_* for exponential distribution.

## Discussion

Protistan cell tracking presents a considerable technological challenge. Consequently, little is known about their chemosensory abilities and few mathematical analyses of their movement are available. The majority of the studies involving micro-protists used species with large cells, such as ciliates [4], [8], [33], species that were easily cultivable (e.g., dinoflagellate *Oxyrrhis*, [9]) or important model organisms (a volvocean green alga, *Chlamydomonas*, [17], [34]) and constitute a rather small body of knowledge compared to animal species. Among protists, nearly all of the species examined were flagellates with documented movement patterns varying from smooth helical swims [4], [9] to those with frequent tumbling, oscillations and reversals of direction [11], [12], [34]. Of these studies, fewer still mathematically characterized movement trajectories. Nonetheless, some of the studies postulated that individual flagellated cells follow a variety of search movement patterns: from Lévy [33] to a standard random walk [34], and their combinations when the environment of the cell changes, at least in laboratory settings. However, such methods have not yet been applied to the movement of either sexual or vegetative diatom cells.

The results of our study showed that male gametes of *T. fasciculata* do not move toward the female gametangia/gametes even while they are in close proximity, and their movement is similar to when female gametes are absent. Based on these findings, we postulate that the male gametes of *T. fasiculata* do not use chemical cues to locate and move towards female gametes. The directional movement of male towards female gamete was also apparently undetectable in *Pseudostaurosira trainorii* ([20] p. 7. point 8, though a somewhat contradictory statement may be found on p. 12), though the movement type(s) was not investigated mathematically. *Tabularia fasciculata* nevertheless probably has the chemosensory ability to detect the presence of a potential mate as they only undergo gametogenesis in the presence of a sex-compatible clone (i.e., not in unisexual control wells). Consequently, by the time male gametes are liberated from the gametangia, male gametes “know” that the females are present in the local vicinity. The precise location of female gametes however remains “unknown” to male gametes, possibly due to the lack of a guiding gradient of chemical cues [11], [12]. Several soil amoeba species also demonstrated a random search pattern in their gradient-free environment [35]. Under such circumstances random movement may indeed be an appropriate strategy for maximizing the probability of encounter between motile male and stationary female targets. Direct contact between male and female gametes is necessary to initiate mate recognition and fertilization.

Despite all the difficulties, relatively high fertilization success was observed in *T. fasciculata* (55–80% [25]). Similar success rates have been shown in the raphid diatom *Pseudo-nitzschia multiseries* (*in vitro*), the only other diatom for which fertilization success has been quantified (56–70% [14]), and in natural populations of intertidal red algae (29–91% [36]) as well as several externally fertilized marine invertebrate and vertebrate species (0–90% [37] which were strongly dependent on water turbulence). The question still remains as to how such high fertilization rates can be achieved if males cannot detect the location of female gametes even when in very close proximity.

Random walk patterns have been investigated in a large variety of organisms (including a few flagellated and non-flagellated protists) and the results show that individuals may use a variety of walk types in pursuit of a target resource [4], [9], [34], [38] and that the optimal search pattern is dependent upon characteristics of the resource [5], [28]; scarce resources seem to be often exploited by application of the Lévy walk. Interestingly, in the case of *T. fasciculata*, the resource (stationary female gamete), might be considered scarce because it typically is outnumbered by male gametes (ratio of 1∶2–8 or more [18], [25]). Indeed, the distribution of male gamete move lengths was found to be consistent with a truncated power law distribution with an exponent of 2.34. This would be consistent with a Lévy walk search pattern, but the range of move lengths in the tail (spanning slightly more than one order of magnitude) is too narrow for the Lévy walk properties to emerge. Therefore search patterns would be best described as Brownian motion. It thus appears that the theoretically optimal strategy is not employed by *T. fasciculata* male gametes.

A number of factors may explain this apparent discrepancy. First, the means of propulsion in male gametes (extension and retraction of pseudopodia with a finite maximum length and rate of production) may not allow for the occasional very long displacements necessary for Lévy properties to emerge. Second, to attain a high fertilization rate, *T. fasciculata* may have adopted a strategy based on sexualisation ratio rather than most efficient search patterns. Third, the laboratory setting used may not fully replicate the natural conditions under which syngamy occurs. Vegetative cells and gametangia of *T. fasciculata* are normally attached to a substratum (macroalgae in the case of our clones), thus within an environment with relatively slow moving water (laminar flow layer with low Reynolds numbers [39]), even though it is contained within the generally turbulent intertidal environment. Under these conditions, it is possible that some advection occurs (an occasional stronger wave, for example), and that this contributes to the redistribution of male gametes. This may increase fertilization success, especially when sessile female gametes are not lost by being advected away (e.g., [40]).

Male gamete motility observed in *T. fasciculata* is exceptional among diatoms. The novel means of gamete propulsion may represent a response by this and three other taxa [18], [19], [20], [41] to pressure(s) imparted on immotile gametangia subsequent to the loss of flagellated sperm characteristic of ancestral centric diatoms. In this context there seems to be somewhat of a mystery with *T. fasciculata* in that it possesses a very novel form of gamete propulsion, yet appears to utilise an indiscriminate mate-search pattern. Diatom genera with visibly motile male gametes (*Tabularia*, *Pseudostaurosira* and *Ulnaria*) belong to derived “core” araphids [42], [43]. Unfortunately, details of sexual reproduction have not yet been reported for any member of the “basal” araphids, the Rhaphoneidaceae-Plagiogrammaceae-Asterionellopsis clade, and so the ground-state of male-gamete behaviour for all the pennates remains unknown. Thus it is not possible at this time to determine whether the pseudopodia-generated male gamete motility is an evolutionary novelty specific to these “core”, yet distantly related, araphid genera, compensating for immotile parents and their gametangia, or a novel use of a structure (pseudopodia) that is well dispersed among many protists and animals [19].

Further study is required to determine whether the movement strategy employed by *T. fasciculata* male gametes is indeed an optimal approach. Varying the density of male and/or female gametes and comparing fertilization success rate after a certain time interval to a simulated random walker’s could confirm whether the random walk pattern is an overall effective search strategy or is abandoned in favor of a different search type. Field studies of fertilization success for other araphid diatom species utilizing alternate mate-search strategies (e.g., reliance on water turbulence with limited amoeboid motility) would also provide insight into the relative effectiveness of the unusual gamete movement in *T. fasciculata*. Nonetheless, our study along with others demonstrate that mathematical analyses of random walks are useful tools informing on the behaviour of individuals and on otherwise indiscernible aspects of protistan ecology.

## Supporting Information

Video S1 **Movement of male gamete in absence of receptive female.** Video rendered from 50 frames acquired every 15 seconds; actual time interval 12.5 minutes. Red circle indicates starting position of male gamete whose trajectory was measured for the study. A long pseudopodia in the target gamete can be seen at approximately the 2 second point in the video. Other male gametes in the field of view demonstrate typical spinning and shuffling movements.
(AVI)

Video S2 **Movement of male gamete in presence of receptive female.** Video rendered from 50 frames acquired every 15 seconds; actual time interval 12.5 minutes. Red circle indicates starting position of male gamete whose trajectory was measured for the study. Inset shows position of receptive female in 10x field of view (not visible in 20x field of view of video).
(AVI)
